# Channel semantic mutual learning for visible-thermal person re-identification

**DOI:** 10.1371/journal.pone.0293498

**Published:** 2024-01-19

**Authors:** Yingjie Zhu, Wenzhong Yang

**Affiliations:** 1 College of Software, Xinjiang University, Urumqi, China; 2 Xinjiang Multilingual Information Technology Laboratory, Xinjiang University, Urumqi, China; Nanchang University, CHINA

## Abstract

Visible-infrared person re-identification (VI-ReID) is a cross-modality retrieval issue aiming to match the same pedestrian between visible and infrared cameras. Thus, the modality discrepancy presents a significant challenge for this task. Most methods employ different networks to extract features that are invariant between modalities. While we propose a novel channel semantic mutual learning network (CSMN), which attributes the difference in semantics between modalities to the difference at the channel level, it optimises the semantic consistency between channels from two perspectives: the local inter-channel semantics and the global inter-modal semantics. Meanwhile, we design a channel-level auto-guided double metric loss (CADM) to learn modality-invariant features and the sample distribution in a fine-grained manner. We conducted experiments on RegDB and SYSU-MM01, and the experimental results validate the superiority of CSMN. Especially on RegDB datasets, CSMN improves the current best performance by 3.43% and 0.5% on the Rank-1 score and mINP value, respectively. The code is available at https://github.com/013zyj/CSMN.

## 1 Introduction

Person re-identification (ReID) [1] is a technology that employs computer vision algorithms to locate and retrieve a pre-defined individual from non-overlapping camera views. Previous studies [2–7] have mainly focused on ReID in visible light, capturing all images of a person with visible light cameras. However, visible light cameras may not capture a person’s appearance at night. As a result, VI-ReID [8] is proposed.

Compared to single-modality ReID, VI-ReID faces the problem of intra-class variations, such as illumination and occlusion, and the challenge of significant modality discrepancy. Therefore, VI-ReID is more challenging. Currently, common methods for VI-ReID mainly include the following aspects: On the one hand, modality-invariant features are extracted to address the cross-modality problem [9, 10]. However, modality-invariant features are frequently difficult to ensure quality, leading to the loss of information in pedestrian image representations. On the other hand, using GAN methods for cross-modality transformation [11–14] can convert cross-modality matching problems into within-modality matching tasks to improve retrieval accuracy. However, such methods inevitably increase the computational complexity of the model and introduce noise, resulting in poor model performance. In addition, some work has been devoted to improving the performance of metric learning methods [15–18]. But the above methods only learn the sample distribution at the instance level and lack handling of outlier samples.

**Fig 1:**
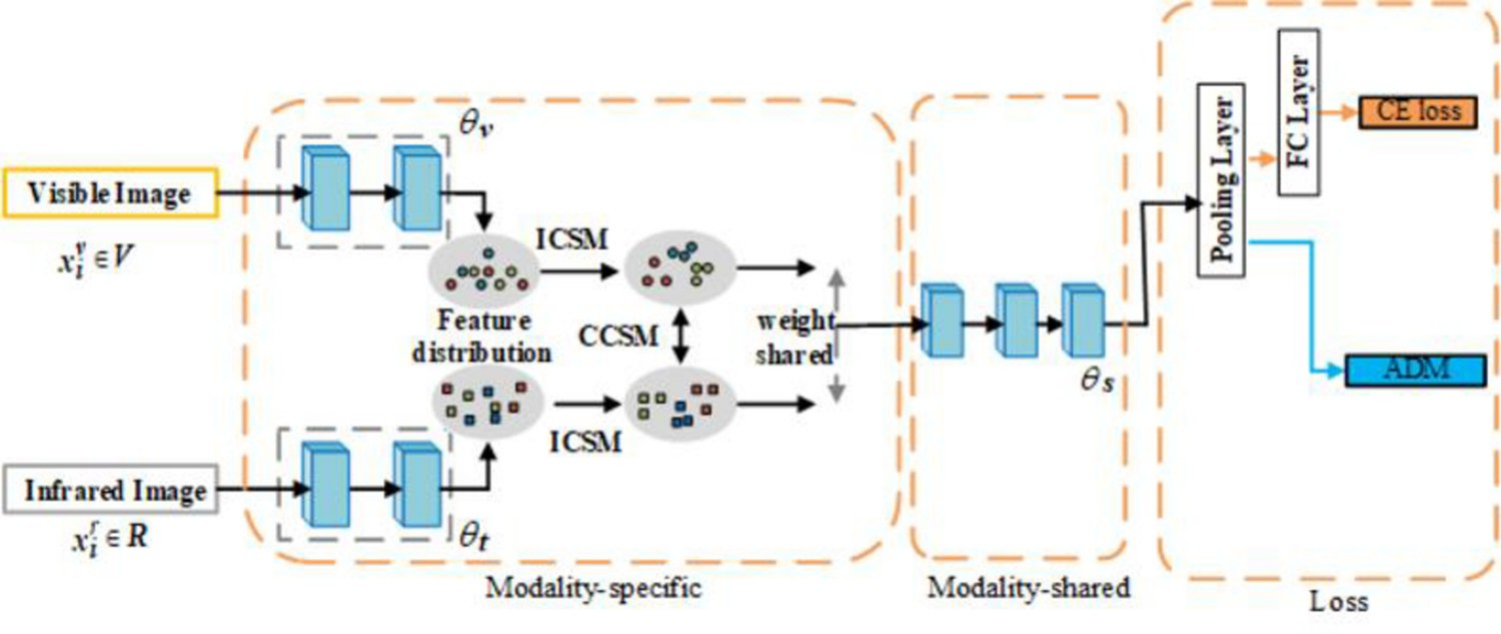
The figure shows the overall structure of the model.

To reduce the discrepancy between the channel semantics within a modality and between modalities, we designed a novel Channel Semantic Mutual Learning Network (CSMN), which simultaneously learns channel semantic consistency from two aspects: Intra-Modality Channel Semantic Mutual Learning(ICSM), which focuses on learning fine-grained information by increasing the similarity of feature distributions between channels, and Cross-Modality Channel Semantic Mutual Learning (CCSM), aiming at learning global information by reducing the distance between feature distributions across modalities. In addition, we proposed a Channel-level Auto-directed Metric Learning loss (CADM) to optimise intra-class and inter-modality feature distributions in a more fine-grained manner. Specifically, our approach reduces intra-class instance discrepancies and aggregates semantic information for the same identity while also narrowing the gap between modalities by strengthening the correlation between semantic information for the same identity across different modalities. Additionally, we designed an auto-guided function to mitigate the generation of noisy samples. Since infrared images cannot be viewed as normal RGB images, we use the gray-to-color method to convert infrared images to colored images. Fig 1 shows the overall structure of the model.

In summary, the main contributions of this paper are:

We propose a channel semantic mutual learning network (CSMN) for VI-ReID that treats modality discrepancy as inter-channel discrepancy and reduces intra-modality channel discrepancy while learning inter-modal channel information to bridge modality discrepancy.

We suggest a channel-level auto-guided double metric loss (CADM) to optimise the sample distribution intra- and inter-modality through multiple aspects, including reducing the intra-class instance differences, strengthening the correlation between the same identity across different modalities, and handling outlier samples.

We have conducted numerous experiments on two benchmarks. Specifically, on SYSU-MM01, CSMN achieves state-of-the-art performance and improves the Rank-1 score of the current best performance on the RegDB dataset by 3.43%.

## 2 Related work

### 2.1 Single-modality ReID

Single-modality ReID attempts to retrieve a specific person from a library of images obtained from different cameras during the day, where the images obtained have the same modalities. Person re-identification (ReID) methods have greatly improved as deep learning technology has advanced. Many methods have focused on building local-based models to fully explore fine-grained features in a person’s images [19–21]. Fu et al. [10] learned local features at different scales using a pyramid structure and eventually obtained multi-scale fused features. Lian et al. [20] designed an attention-aligned network for feature learning that uses channel and spatial attention. Wang et al. [21] proposed a multi-branch network where one branch captures global representations and the other branch focuses on local information. In addition, attention models [22–26] are essential for designing novel network architectures that highlight salient regions and alleviate misalignment to learn robust features.

## 2.2 Visible-infrared person re-identification

VI-ReID is to match and identify the same pedestrian between different cameras, not different modalities. Wu et al. [8] published SYSU-MM01dataset and proposed a model to extract modality-shared person features. Dai et al. [27] suggested that cmGAN reduces the modality differences between visible and infrared images. Thus, dual-stream networks have been widely used to address modality discrepancy problems [16, 28]. Ye et al. [16] proposed a model to address intra-class variation caused by viewpoint non-variation. Ye et al. [28] proposed a novel DDAG learning method for VI-ReID by mining modal contextual cues. However, the methods above focus on reducing modality differences at the instance level. At the same time, this paper aims to learn more discriminative clues at the channel level, enabling semantic consistency between channels.

### 2.3 Metric learning

Metric learning plays a crucial role in inter-sample similarity measures for Re-ID. Ye et al. [16] provided a loss to learning discriminative feature representations using a two-stream network [29]. They also introduced a major constraint to enhance performance [30]. To reduce intra- and cross-modal variation, Hao et al. [31] proposed a network with classification and recognition constraints. Zhao et al. [32] introduced the hard pentaplet loss to improve VI-ReID performance. Wu et al. [33] designed a novel loss for focal modality-aware that guides inter-modal similarity learning with intra-modal similarity. However, the above methods only use the Euclidean metric, which cannot learn modality-shared discriminative features from multiple perspectives. And ignore the impact of noisy samples on model performance.

## 3 Method

In this section, as shown in Fig 1, we introduce CSMN, which consists of three parts: Modality-specific, Modality-shared and Loss, with the Modality-specific containing two essential elements: 1) Intra-modality Channel Semantic Mutual Learning (ICSM), which reduces differences between instances by learning semantic information among channels of instances within the same modality. 2) Cross-modality Channel Semantic Mutual Learning (CCSM), which learns the relationships between channels of different modalities and aggregates semantic information of the same identity at the channel level. Then the features learned from different branches go through Modality-shared for further feature learning. In terms of metric learning, we design Channel-level Auto-guided Double Metric loss (CADM), which optimizes the distribution of samples within and between modalities.

### 3.1 Intra-Channel Semantic Mutual Learning

**Fig 2:**
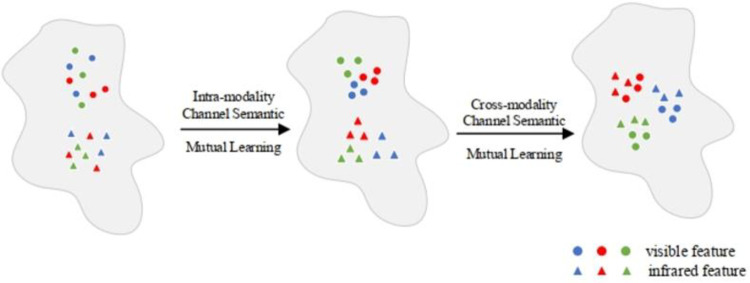
Channel semantic mutual learning.

RGB image channels contain different semantic information and have certain correlations. As depicted in Fig 2, modality-specific features are extracted by specific feature layers. As visible light and infrared images are captured based on different imaging principles, modality-specific features correspond to different semantic information for the same identity. Since infrared images are obtained based on the temperature distribution on the surface of objects, they cannot be treated as ordinary three-channel images. In this paper, we attribute the differences between modalities to differences between channels. Hence, the key to this problem is to ensure the identity correlation of channel features and reduce semantic changes between channels. Since the extended three-channel infrared image exhibits heterogeneity in the R/G/B channels, we aim to train the network to learn R/G/B channel distributions similar to those of visible images. To reach this goal, we made an Intra-Modality Channel Semantic Mutual Learning (ICSM) module, as shown in Fig 2, which uses the colours red, blue, and green to show how similar the channel feature distributions are to each other. Our method focuses on maximizing the intra-modality channel-level semantic consistency within each modality. We represent channel-level consistency as the logical distribution similarity between channel features. It can be formulated as follows:

$$L_{ICMC}(\theta_{v},\theta_{t})=\frac{1}{2}*\left[(\sum_{i=1}^{N}f_{i}^{R_{v}}.log\frac{f_{i}^{R_{v}}}{f_{i}^{G_{v}}+f_{i}^{R_{v}}}+f_{i}^{R_{t}}.log\frac{f_{i}^{R_{t}}}{f_{i}^{G_{t}}+f_{i}^{R_{t}}})+(\sum_{i=1}^{N}f_{i}^{B_{v}}.log\frac{f_{i}^{B_{v}}}{f_{i}^{G_{v}}+f_{i}^{B_{v}}}+f_{i}^{B_{t}}.log\frac{f_{i}^{B_{t}}}{f_{i}^{G_{t}}+f_{i}^{B_{t}}})+(\sum_{i=1}^{N}f_{i}^{B_{v}}.log\frac{f_{i}^{B_{v}}}{f_{i}^{R_{v}}+f_{i}^{B_{v}}}+f_{i}^{B_{t}}.log\frac{f_{i}^{B_{r}}}{f_{i}^{R_{t}}+f_{i}^{B_{t}}})\right]$$ (1)

where *L*_ICMC_ represents the semantic consistency between the three channels, and ICSM aims to minimize the semantic difference between channels. To achieve the above goals, the following formula is used to optimize the parameters *θ*_*v*_ and *θ*_*t*_:

$$\left({\hat{\theta }}_{v}{\hat{\theta }}_{t})=arg\;min(L_{ICMC}(\theta_{v},\theta_{t})\right)$$ (2)

### 3.2 Cross-Modality Channel Semantic Mutual Learning

In addition, to reduce the differences between modalities, we propose the Cross-Modality Channel Semantic Mutual Learning (CCSM) method, which aims to maximize the inter-modality channel semantic consistency. This method uses inter-modality semantic consistency to aggregate features from different modalities under the same identity. We further reduce the inter-modality channel semantic differences based on the modality-specific semantic consistency features. Since the modality-specific extractors *θ*_*v*_ and *θ*_*t*_ extract features within each modality, the extracted modality-specific features are independent. The following formula can represent the features of each modality:

$$C_{v}=(1/\sum_{i=1}^{S_{v}}w_{i}^{v})*\sum_{i=1}^{S_{v}}w_{i}^{v}*f_{i}^{v}$$ (3)

$$C_{t}=(1/\sum_{i=1}^{S_{t}}w_{i}^{t})*\sum_{i=1}^{S_{t}}w_{i}^{t}*f_{i}^{t}$$ (4)

where *S*_*t*_ and *S*_*v*_ represent the number of samples in the infrared and visible images and represent the weights of the i-th feature vector in different modalities, which are adjusted to reduce the influence of outliers. *C*_*v*_ and *C*_*t*_ are batch-computed. CCSM aims to learn semantic information between modalities rather than identity information. Using metric learning enables the alignment of the distance between *C*_*v*_ and *C*_*t*_, and the features *θ*_*v*_ and *θ*_*t*_ will have more semantic consistency information. In CCSM, the goal is to maximize the inter-modality semantic consistency between visible and infrared image features:

$$L_{ccsm}=L(\theta_{v},\theta_{t}){=\|C_{v}-C_{t}\|}^{2}$$ (5)

**Fig 3:**
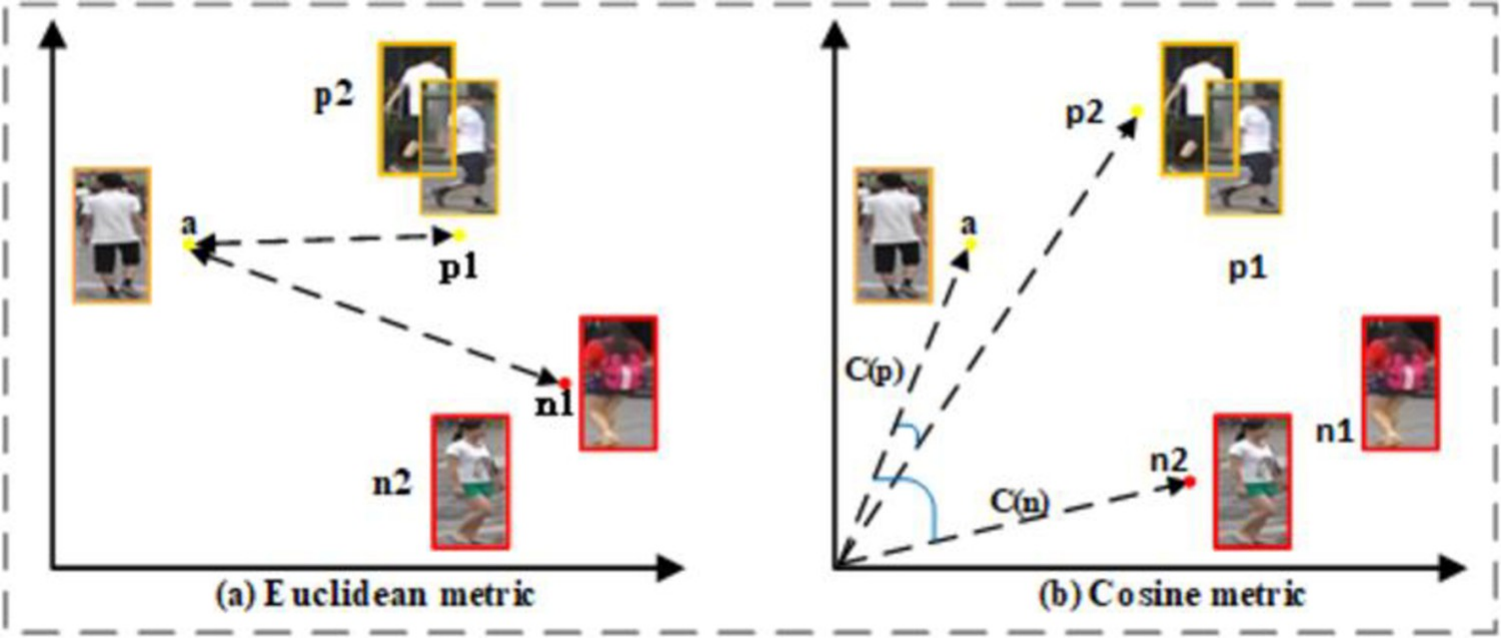
The diagram depicts the single and double metric learning methods. Where *C(p)* and *C(n)* represent the cosine values between the positive and negative samples.

Fig 3 shows the collaborative processing process of ICSM and CCSM. The combination of the two can not only reduce the differences between instances of the same identity within each modality and improve the matching accuracy of the same identity between modalities.

### 3.3 ADM

Most existing metric learning methods are at the instance level, a coarse-grained learning method that is also vulnerable to the influence of noisy samples. To learn fine-grained features and reduce the impact of noisy samples on the feature space, we propose channel-level auto-guided double metric loss (CADM). We obtain semantically consistent features *f*^*v*^ and *f*^*r*^ from the modality-specific extractors *θ*_*v*_ and *θ*_*t*_ respectively. Then we use a weight-shared feature extractor *θ*_*s*_ to obtain rich semantic features [$f_{i}^{R_{v}},f_{i}^{G_{v}},f_{i}^{B_{v}}$], $[f_{i}^{R_{t}},f_{i}^{G_{t}},f_{i}^{B_{t}}]\in R^{B\times C\times H\times W}$. Since different metric learning methods will learn different the hardest samples. For the Euclidean metric, *p*1 and *a* in Fig 3(A) are the pair of positive samples, and n1 and a are the pair of negative samples, but for the cosine metric in Fig 3(B), *p*2 and *a* are only the pair of positive samples, and n2 and a are the pair of negative samples. So, to learn the sample distribution from multiple perspectives, we propose a double metric loss(DM), which introduces a cosine metric that takes the direction of the feature vector into account based on the Euclidean metric:

$$L_{e}{=\|f_{i}^{R_{v}}-f_{i}^{R_{t}}\|}^{2}+{\left\|f_{i}^{G_{v}}-f_{i}^{G_{t}}\right\|}^{2}+{\left\|f_{i}^{B_{v}}-f_{i}^{B_{t}}\right\|}^{2}$$ (6)

$$L_{c}=(1-\frac{f_{i}^{R_{v}}∙f_{i}^{R_{t}}}{\left\|f_{i}^{R_{v}}\|\|f_{i}^{R_{t}}\right\|})+(1-\frac{f_{i}^{G_{v}}∙f_{i}^{G_{t}}}{\left\|f_{i}^{G_{v}}\|\|f_{i}^{G_{t}}\right\|})+(1-\frac{f_{i}^{B_{v}}∙f_{i}^{B_{t}}}{\left\|f_{i}^{B_{v}}\|\|f_{i}^{B_{t}}\right\|})$$ (7)

Additionally, to deal with noisy samples, we introduce an auto-guided function. Specially, we utilize the Euclidean metric to calculate the similarity between samples and construct the corresponding similarity matrix. We initially extract the positions of all positive and negative samples from the distance matrix using the Euclidean metric to generate a position mask. The position mask calculates the distances between each sample in the distance matrix. All sample distances are then combined using the proposed auto-guided function. The following is the auto-guided function.

$$P(x)=\left\{ \begin{array}{c} 0,\;x<0\\ \frac{x}{2d}+\delta ,\;0<x<2d\\ x,\;x>2d \end{array}\right.$$ (8)

where d is the constant slope that controls the auto-guided function. *δ* is a very small constant that ensures the function value is greater than zero.

The CADM can finally be expressed as:

$$L_{cadm}=L_{e}+b∙L_{c}+c∙L_{p}$$ (9)

Where *c* are the auto-guided function loss coefficients of L_*p*_.Therefore, the final expression of the loss function is as follows:

$$L_{total}=L_{ICMC}+L_{ccsm}+L_{cadm}$$ (10)

## 4 Experiment

### 4.1 Experimental settings

#### 4.1.1 Dataset and setting

We evaluate the performance of our proposed approach on the VI-ReID task through experiments on two widely used benchmark datasets, SYSU-MM01 [8] and RegDB [34]. The SYSU-MM01 dataset, the largest VI-ReID dataset, comprises four visible and two near-infrared cameras. The training set consists of 22,258 visible images and 11,909 thermal images of 395 individuals. The test set has 96 distinct identities, with 3,803 thermal images used as queries and 301 visible images used as galleries. We used single-shot outdoor and indoor search modes in our experiments. The dataset’s configuration details can be found in [35]. The RegDB dataset consists of images captured by one visible camera and one far-infrared camera. It contains 412 identities, each represented by 20 images (10 visible and 10 infrared) per person. According to the current VI-ReID settings [36], 206 identities are chosen randomly for training, and the remaining 206 identities are allocated to the test set.

#### 4.1.2 Evaluation metrics

To assess the performance of our method, we use cumulative matching characteristics (CMC), mean average precision (mAP), and mean inverse negative penalty (mINP) [36]. mAP evaluates the retrieval system’s performance when a gallery set contains multiple matched images. CMC measures the probability that the top-ranked retrieval results have the correct image of the person. mAP evaluates the retrieval system’s performance when a gallery set contains multiple matched images. Furthermore, mINP considers the most difficult match to calculate the amount of work for inspectors.

#### 4.1.3 Implementation details

We use CAJL [37] as the baseline network. The pre-trained weights of ImageNet are used to initialize the network parameters. We employ a PK sampling design with P = 8 and K = 4 parameters. We use zero-padded, randomly cropped images (288 × 144) as training data to supplement the original dataset. The SGD optimizer is used during the optimization process’s learning phase. The learning rate is reduced from its initial value of 0.1 after 20 and 50 iterations. There are 100 training epochs in total. All tests were performed on an Nvidia 3090 GPU with PyTorch 1.6 and cuda11.0.

### 4.2 Ablation study

To verify the effectiveness of ICSM, CCSM, DM, and CADM, we conducted detailed experiments on the RegDB and SYSU-MM01dataset.

#### 4.2.1 Effectiveness of Intra-Channel Semantic Mutual Learning (ICSM)

As shown in Table 1, taking the visible to infrared mode as an example, based on the Base model, using only *L*_*ICSM*_ achieved a Rank-1 score of 86.5% and an mAP score of 77.25%. This improved the Rank-1 score of the baseline model by 1.47%. The experimental results show that ICSM can learn fine-grained features among channels within a modality, reducing the differences between channels within the same modality.

**Table 1:** Ablation experiments regarding *L*_*ICSM*_ and *L*_*CCSM*_ were studied on the RegDB dataset, where Base refers to CAJL [34].

Methods			RegDB(Visible to Infrared)			RegDB(Infrared to Visible)		
Base	*L*_*ICSM*_	*L*_*CCSM*_	Rank-1	mAP	mINP	Rank-1	mAP	mINP
√			85.03	79.14	65.33	84.75	77.82	61.56
√	√		86.50	77.25	64.89	85.67	76.02	59.34
√		√	88.01	79.99	65.59	85.98	77.08	62.42
√	√	√	88.11	80.06	65.75	86.21	77.12	62.54

#### 4.2.2 Effectiveness of Cross-Modality Channel Semantic Mutual Learning(CCSM)

Unlike ICSM, CCSM learns global information between modalities to aggregate samples of the same identity across modalities. As shown in Table 1, *L*_*CCSM*_ represents the loss between modalities. Taking the visible-to-infrared mode as an example, using only *L*_*CCSM*_ on the Base model increased the Rank-1 score and mAP score by 2.98% and 0.85%, respectively. Additionally, we observed that using both *L*_*CCSM*_ and *L*_*ICSM*_ on the Base model further improved the model’s performance. Specifically, compared to Base + *L*_*ICSM*_, the Rank-1 and mAP scores improved by 1.61% and 2.81%, respectively. The Rank-1 score of Base + *L*_*CCSM*_ was improved by 0.1%, and the mAP score was improved by 0.07%. From the experimental results, CCSM can effectively reduce the differences between modalities, and combining ICSM and CCSM further enhances the model’s performance.

#### 4.2.3 Effectiveness of Double Metric Loss (DM)

As shown in Table 2, we conducted a series of experiments on the methods based on Euclidean and cosine metrics to demonstrate that the double metric consisting of Euclidean and cosine metrics can effectively improve the baseline performance. It should be noted that our experiments were conducted based on Base+*L*_*ICSM*_+*L*_*CCSM*_. Baseline1 only uses the Euclidean metric, whereas Baseline2 only uses the cosine metric. The DM-based baseline model outperformed Baseline1 on the RegDB dataset by 0.53% and 0.39% on Rank-1 and mAP, respectively, and by 0.41% and 0.05% on the SYSU-MM01 dataset. The experimental results show that DM can learn the feature distribution from multiple perspectives in a fine-grained manner, thereby improving the performance of the model.

**Table 2:** DM ablation experiment.

Method	Metric		RegDB		SYSU-MM01	
	Euclidean	cos	Rank-1	mAP	Rank-1	mAP
Baseline1	√		88.11	80.06	69.88	66.89
Baseline2		√	87.86	79.77	69.95	66.34
**DM(ours)**	√	√	**88.20**	**80.83**	**70.29**	**66.94**

#### 4.2.4 Effectiveness of Channel-level Auto-guided Double Metric Loss (CADM)

We compared our proposed method with commonly used loss functions, as shown in Table 3, and found that CADM outperformed other loss functions, specifically CELoss by 5.09% at Rank-1 and TripletLoss by 4.19% on the RegDB dataset. In addition, Rank-1 score over 1.02% of DM. On the SYSU-MM01 dataset, the Rank-1 score of the proposed method is 0.92% higher than that of DM. Experimental results show that CADM can effectively handle abnormal samples.

**Table 3:** Comparison of our proposed loss with other common loss functions.

Loss	RegDB		SYSU-MM01	
	Rank-1(%)	mAP(%)	Rank-1(%)	mAP(%)
CenterLoss	83.56	77.96	68.01	65.98
CELoss	84.13	78.34	68.94	66.13
TripletLoss	85.03	79.14	69.88	66.89
DM(Ours)	88.20	80.83	70.29	66.94
**CADM(Ours)**	**89.22**	**83.57**	**71.21**	**67.68**

### 4.3 Parameter analysis

**Fig 4:**
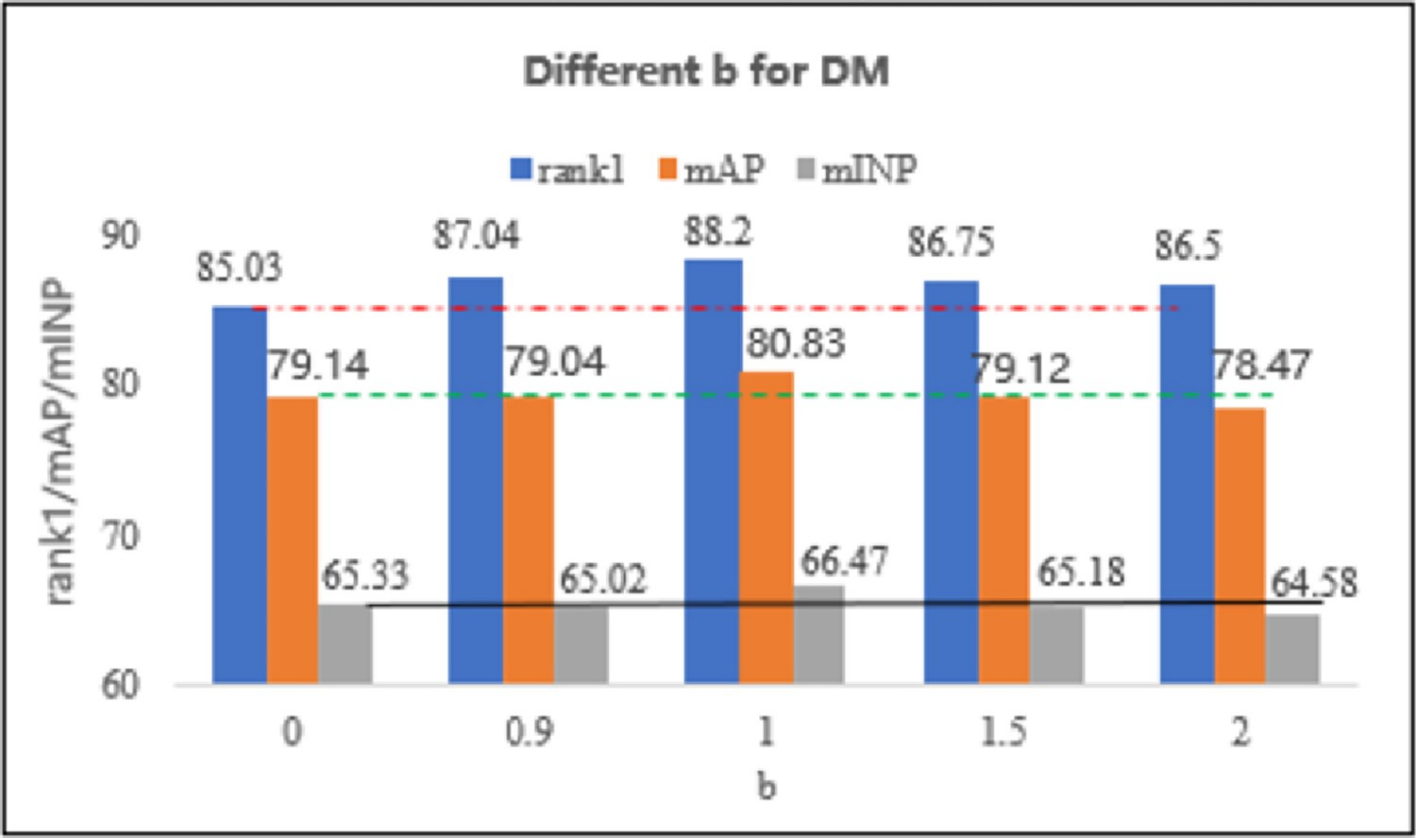
The figure shows the effect of variation in b on the DM performance on RegDB. Rank-1 is represented by the long blue bar, mAP by the long orange bar, and mINP by the long gray bar. The red "—" line represents the rank1 baseline, the green "—" line represents the mAP baseline, and the black "-" line represents the mINP baseline.

Furthermore, we examined different b’s effects on DM on the RegDB dataset. As shown in Fig 4, b = 0 is equivalent to using only the Euclidean metric, and the performance of DM gradually improves as b increases. When b = 1, DM performs optimally; however, as b increases, DM’s performance decreases. This indicates that the two metrics have an equal impact on model performance, demonstrating their complementarity.

As shown in Table 4, on the RegDB and SYSU-MM01 datasets, we tested the effect of different weights *c* on the loss of the auto-guided function. When *c* is small, experimental results show that CADM performance is poor, even worse than DM performance. The weight coefficients are so small that the model parameters do not converge sufficiently. The CADM’s performance is optimal when the weight coefficient *c* is set to 1. In this case, CADM outperforms DM by 1.02%/2.74% on dataset RegDB on Rank-1/mAP and 0.92%/0.74% on dataset SYSU-MM01. When *c* exceeds 1, the CADM’s performance decreases rather than increases. One possibility is that it only amplifies the gradient when it is very large while the model parameters have already been optimized to their maximum.

**Table 4:** The effect of different values of c on the performance of CADM on the RegDB and SYSU-MM01 datasets.

Methods	c	RegDB			SYSU-MM01		
		Rank-1	mAP	mINP	Rank-1	mAP	mINP
Base	-	85.03	79.14	65.33	69.88	66.89	53.61
DM	0	88.20	80.83	66.02	70.29	66.94	54.07
CADM	0.5	86.95	78.89	64.56	68.32	64.96	51.83
CADM	0.9	88.38	80.87	67.40	70.34	66.92	54.02
**CADM**	**1**	**89.22**	**83.57**	**65.93**	**71.21**	**67.68**	**54.12**
CADM	1.25	88.59	79.77	66.16	70.65	67.01	53.97
CADM	1.5	86.26	79.34	65.84	68.47	65.70	51.93

### 4.4 Visualization analysis

**Fig 5:**
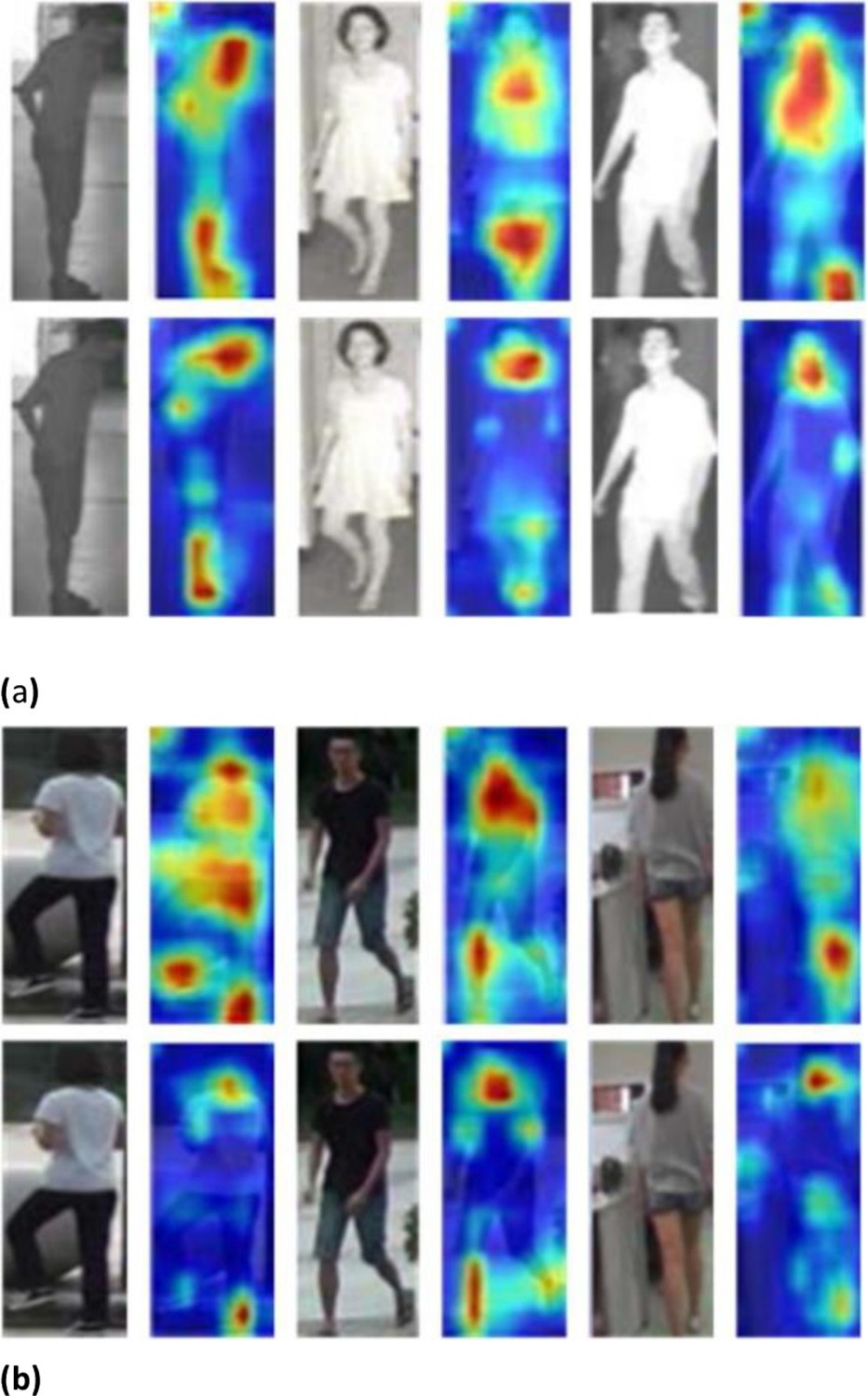
Heat maps extracted by the baseline network (CAJL) and CSMN are displayed on top and bottom, respectively. Note that the pedestrian images is similar but not identical to the original image and is therefore for illustrative purposes only. (a) Comparison of heat maps extracted by the DCMN and the baseline network (CAJL) in infrared modality. (b) Comparison of heat maps extracted by the CSMN and the baseline network (CAJL) in visible modality.

To demonstrate the effectiveness of our proposed method more intuitively, we use heat maps to display the features learned from pedestrian images. The heat maps of pedestrian images in different modalities are shown in Fig 5(A) and 5(B), respectively. The heat map obtained from the CSMN below focuses more on identity-related information than the heat map obtained from the baseline (CAJL [37]) network above, as shown in the figure. This suggests that the CSMN is not particularly sensitive to some distressing information (light, occlusion, etc.). As a result, the model has a high degree of generalizability.

### 4.5 Comparison to the state-of-the-art methods

We compared CSMN with the existing state-of-the-art VT-ReID methods on two benchmark datasets. Tables 5 and 6 show the detailed results for different evaluation metrics.

**Table 5:** Comparison to the state-of-the-art methods on the RegDB dataset.

Method	Venue	Visible to Infrared			Infrared to Visible		
		Rank-1	mAP	mINP	Rank-1	mAP	mINP
Zero-Pad [8]	ICCV-17	14.80	15.95	-	16.63	17.82	-
HCML [16]	AAAI-18	24.44	20.08	-	21.70	22.24	-
HSME [17]	AAAI-19	50.85	47.00	-	50.15	46.16	-
*D*^2^RL [38]	CVPR-19	43.40	44.10	-	-	-	-
AlignGAN [13]	ICCV-19	57.90	53.60	-	56.30	53.40	-
Hi-CMD [39]	CVPR-20	70.93	66.04	-	-	-	-
AGW [36]	arXiv-20	70.05	66.37	50.19	70.49	65.90	51.24
DDAG [28]	ECCV-20	69.34	63.46	49.24	68.06	61.80	48.62
HAT [40]	TIFS-20	71.83	67.56	-	70.02	66.30	-
GMRN [41]	ICIP-21	78.25	71.00	-	-	-	-
MCLNet [42]	ICCV-21	80.31	73.07	57.39	75.93	69.49	52.63
CAJL [37]	ICCV-21	85.03	79.14	65.33	84.75	77.82	61.56
SCFNet [43]	CVPR-22	85.79	81.91	-	86.33	82.10	-
DML [44]	TCSVT-22	77.60	84.30	-	77.00	83.60	-
DSCNet [45]	TIFS-23	85.39	77.30	-	83.50	75.19	-
**CSMN**	**Ours**	**89.22**	**83.57**	**65.93**	**86.89**	**82.34**	**63.12**

**Table 6:** Comparison to the state-of-the-art methods on the SYSU-MM01 dataset.

Method	Venue	All Search			Indoor Search		
		Rank-1	mAP	mINP	Rank-1	mAP	mINP
Zero-Pad [8]	ICCV-17	14.80	15.95	-	20.58	26.92	-
HCML [16]	AAAI-18	14.32	16.16	-	24.52	30.08	-
cmGAN [27]	IJCAI18	26.97	27.80	-	31.63	42.19	-
HSME [17]	AAAI-19	20.68	23.12	-	-	-	-
*D*^2^RL [38]	CVPR-19	28.90	29.20	-	-	-	-
AlignGAN [13]	ICCV-19	42.40	40.70	-	45.90	54.30	-
AGW [36]	arXiv-20	47.50	47.65	35.30	54.17	62.97	59.23
DDAG [28]	ECCV-20	54.75	53.02	39.62	61.02	67.98	62.61
HAT [40]	TIFS-20	55.29	53.89	-	62.10	69.37	-
HCTri [46]	TMM20	61.68	57.51	39.54	63.41	68.17	64.26
GMRN [41]	ICIP-21	57.67	54.88	-	-	-	-
MCLNet [42]	ICCV-21	65.40	61.98	47.39	72.56	76.58	72.10
CAJL [37]	ICCV-21	69.88	66.89	53.61	76.26	80.37	76.79
DML [44]	TCSVT-22	62.20	49.60	-	66.40	60.00	-
DLRL [47]	TIP-22	63.04	60.58	-	67.95	52.12	-
FMCNet [48]	TIP-22	66.34	62.51	-	68.15	74.09	-
**CSMN**	**Ours**	**71.21**	**67.68**	**54.12**	**77.32**	**81.56**	**77.12**

As shown in Table 5, on the RegDB dataset. SCFNet [45] also designs loss functions to reduce the impact of outlier samples on the spatial features and achieves excellent retrieval accuracy. However, our method achieves better results. Specifically, the proposed method outperforms SCFNet by 3.43% in Rank-1 and 1.66% in mAP scores. For the SYSU-MM01 dataset, as shown in Table 6, CSMN achieves state-of-the-art performance. CSMN outperforms the CAJL [37] by 1.33% in Rank-1 and 0.51% in mINP in the more complicated full search mode. To alleviate the strict constraints of traditional triplet loss, the HCTri [39] method, which also improves the loss function, proposes hetero-center triplet loss. Our proposed CSMN, on the other hand, outperforms HCTri on Rank-1 and mAP by 9.53% and 10.17%, respectively. These findings imply that CSMN can effectively reduce the differences between modalities and channels within a modality. On the other hand, CADM can learn the sample distribution in a more fine-grained manner and deal with outlier samples.

## 5 Conclusion

This paper proposes a CSMN framework for visible-thermal person re-identification, which considers cross-modality differences as differences between channels. We reduce the differences between channels in two aspects: On the one hand, we propose ICSM, which learns fine-grained features among channels within a modality to maximize the consistency between channels and minimize the differences between them. On the other hand, we propose CCSM, which learns global channel features between modalities to aggregate samples of the same identity across modalities. In addition, to better optimize the sample distribution between and within modalities, we propose CADM. Unlike methods that learn sample distribution at the instance level, our method fully exploits the advantages of channel consistency to learn the sample distribution in a more fine-grained manner. Moreover, we use an auto-guided function to reduce the generation of outlier samples. Our experiments on two benchmark datasets indicate that CSMN outperforms the existing state-of-the-art methods for VI-ReID.
